# Genetic characterization of clinical and environmental *Vibrio parahaemolyticus* from the Northeast USA reveals emerging resident and non-indigenous pathogen lineages

**DOI:** 10.3389/fmicb.2015.00272

**Published:** 2015-04-07

**Authors:** Feng Xu, Saba Ilyas, Jeffrey A. Hall, Stephen H. Jones, Vaughn S. Cooper, Cheryl A. Whistler

**Affiliations:** ^1^Department of Molecular, Cellular and Biomedical Sciences, University of New HampshireDurham, NH, USA; ^2^Graduate Program in Genetics, University of New HampshireDurham, NH, USA; ^3^Northeast Center for Vibrio Disease and Ecology, University of New HampshireDurham, NH, USA; ^4^Department of Natural Resources and the Environment, University of New HampshireDurham, NH, USA

**Keywords:** disease ecology, emergent pathogen, MLSA, Vibriosis, population structure, pathogen evolution, hemolysin

## Abstract

Gastric infections caused by the environmentally transmitted pathogen, *Vibrio parahaemolyticus*, have increased over the last two decades, including in many parts of the United States (US). However, until recently, infections linked to shellfish from the cool northeastern US waters were rare. Cases have risen in the Northeast, consistent with changes in local *V. parahaemolyticus* populations toward greater abundance or a shift in constituent pathogens. We examined 94 clinical isolates from a period of increasing disease in the region and compared them to 200 environmental counterparts to identify resident and non-indigenous lineages and to gain insight into the emergence of pathogenic types. Genotyping and multi-locus sequence analysis (MLSA) of clinical isolates collected from 2010 to 2013 in Massachusetts, New Hampshire, and Maine revealed their polyphyletic nature. Although 80% of the clinical isolates harbored the *trh* hemolysin either alone or with *tdh*, and were urease positive, 14% harbored neither hemolysin exposing a limitation for these traits in pathogen detection. Resident sequence type (ST) 631 strains caused seven infections, and show a relatively recent history of recombination with other clinical and environmental lineages present in the region. ST34 and ST674 strains were each linked to a single infection and these strain types were also identified from the environment as isolates harboring hemolysin genes. Forty-two ST36 isolates were identified from the clinical collection, consistent with reports that this strain type caused a rise in regional infections starting in 2012. Whole-genome phylogenies that included three ST36 outbreak isolates traced to at least two local sources demonstrated that the US Atlantic coastal population of this strain type was indeed derived from the Pacific population. This study lays the foundation for understanding dynamics within natural populations associated with emergence and invasion of pathogenic strain types in the region.

## Introduction

Rare pathogenic variants of *Vibrio parahaemolyticus*, a ubiquitous yet typically harmless estuarine bacterium, can cause human gastric infections most often from the consumption of raw or improperly handled seafood, and wound infections from recreational aquatic activities (Daniels et al., 2000; Scallan et al., 2011). Infections typically occur seasonally during warmer months when total populations of these bacteria rise, and with them, the risk of exposure to an infectious dose of pathogens increases (Parveen et al., 2008). Even so, temperature and total abundance do not fully explain infection trends as some infections occur when water temperatures and abundance of total *V. parahaemolyticus* are low (Zimmerman et al., 2007; Johnson et al., 2012; Jones et al., 2012). Furthermore, recurrent infections and outbreaks have also occurred in cooler waters of the Pacific Northwest (PNW) for decades where pathogens re-emerge each year from among diverse residential populations (Altekruse et al., 2000; Johnson et al., 2010; Paranjpye et al., 2012; Turner et al., 2013; Banerjee et al., 2014). A better understanding of conditions that promote emergence and relative abundance of pathogens is necessary to develop appropriate strategies for disease prevention.

**Figure 1:**
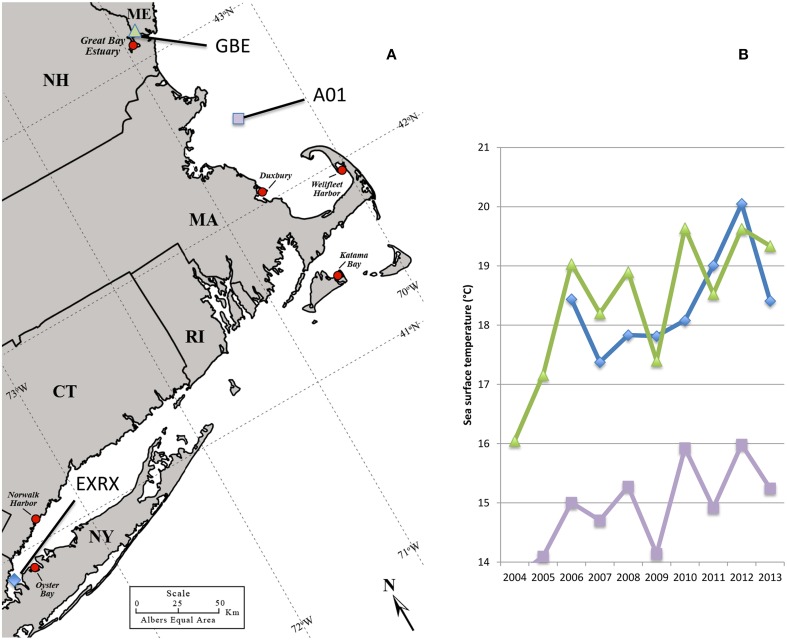
**Regional oyster production areas and ocean temperatures**. Regional oyster harvest and production areas all identified with red circle, some of which have been linked to cases or outbreaks of *V. parahaemolyticus* including: Oyster Bay NY in 1998, 2012, and 2013; Norwalk Harbor CT in 1998, and 2013; Katama Bay MA in 2013; and Duxbury MA in 2013. No closures had occurred at the Great Bay Estuary site in NH or Wellfleet Harbor in MA as of 2013 **(A)**. Ocean buoys used for temperature measurements identified by blue diamond, purple square, and green triangle symbols **(A,B)**. Increasing average monthly sea surface water temperatures during June 2004–2013 at offshore buoys in Long Island Sound (EXRX), the Gulf of Maine (A01), and the Great Bay Estuary (GBE) **(B)**. Map was adapted from the US Department of Agriculture Natural Resource Conservation Service.

Comparatively fewer and sporadic infections have been associated with shellfish harvested from the cooler waters of the Northeastern US. One notable exception was a large multi-state outbreak in 1998, which included oysters harvested from Long Island Sound bordered by New York (NY) and Connecticut (CT) (Figure 1) (CDC, 1999) That outbreak was attributed to a non-indigenous strain from the pandemic clonal complex which are typically serotype O3:K6 and sequence type (ST) 3 and that spread globally from Southeast Asia (CDC, 1999; Depaola et al., 2000; Nair et al., 2007; García et al., 2009; Harth et al., 2009; Martinez-Urtaza et al., 2010; Alikhan et al., 2011). The ensuing low disease incidence in the Northeastern US implied that the regional environmental conditions did not sustain either invasive or endemic pathogenic populations (Mahoney et al., 2010; Ellis et al., 2012). In the last several years, however, reported cases have been increasing in the Northeast, with outbreaks in NY in 2012, and in NY, CT, and Massachusetts (MA) in 2013 (Newton et al., 2014) (Figure 1). This abrupt increase in cases coincided with both warmer than usual ocean temperatures in the region (Figure 1) and the probable Atlantic ecological invasion of a lineage of ST36 strains, which are indigenous to the cooler waters of the PNW (Martinez-Urtaza et al., 2013; Newton et al., 2014). Thus, the emergent disease in the Northeast appears to be unlike other situations under study in the US. Characterizing clinical strains from the region and relating them to native non-pathogens during this period of increased disease incidence could provide insight into how changes in the bacterial population have led to increased disease.

A significant obstacle for the study of emergent pathogenic strains of *V. parahaemolyticus* is our lack of understanding of factors that define virulence and that could be used to detect pathogens within mostly non-pathogenic populations. Few of the diagnostic markers most commonly used to define pathogens are actually implicated in disease, including two hemolysin genes (*tdh* and *trh*) and a horizontally acquired type-three secretion system (T3SS2) (Honda and Iida, 1993; Hiyoshi et al., 2010). Although hemolysins are sufficient for inducing some disease symptoms, they are not necessary for disease in either mice or humans, indicating they are not the only virulence determinant (Nishibuchi et al., 1992; Xu et al., 1994; García et al., 2009; Thongjun et al., 2013; Banerjee et al., 2014). Perhaps more concerning, the abundance of hemolysin-containing strains in the environment often does not correlate with increased incidence of disease, calling into question the ability of these markers to sufficiently predict risk evaluation (Johnson et al., 2012; Jones et al., 2012).

In the absence of a definitive diagnostic marker of virulence, identification of related strains or lineages of pathogens that are known to cause infections would aid in the study of seasonal population dynamics associated with emergence of these pathogenic types. The many commonly applied strain typing tools, including serotyping, rep-PCR, pulse field electrophoresis (PFGE), intergenic spacer region (ISR-1), and restriction fragment length polymorphisms can group similar strains but have some limitations for resolving evolutionary relationships, especially when used individually (Chowdhury et al., 2000, 2004; Nair et al., 2007; Jones et al., 2012; Banerjee et al., 2014; Lüdeke et al., 2014). Multi-locus sequence analysis (MLSA) of conserved housekeeping genes can better define relatedness and recombination among strains (Depaola et al., 2003; Jolley et al., 2004; Thompson et al., 2005; Sawabe et al., 2007; González-Escalona et al., 2008; Ellis et al., 2012; Paranjpye et al., 2012; Martinez-Urtaza et al., 2013; Turner et al., 2013; Banerjee et al., 2014). But the analysis of pathogens alone, in the absence of related non-pathogenic relatives, will not provide a complete picture of evolution of virulence.

Here we combine MLSA, genotyping, and phylogenetic analysis to relate clinical strains with environmental isolates from northern New England. The strains analyzed include 94 clinical isolates from reported cases in three northern New England US states and more than 200 environmental isolates identified from the region since 2007 (Mahoney et al., 2010; Ellis et al., 2012). The study demonstrated that disease-causing strains are genetically diverse and polyphyletic. Some infections were caused by pathogens that are resident, but the strains that caused a steep rise in infections in 2013 are derived from the PNW ST36 population as previously suggested (Martinez-Urtaza et al., 2013; Newton et al., 2014). This study lays the foundation for detailed analysis of conditions that have promoted emergence and invasion of pathogenic types in the region.

## Materials and methods

### Bacterial strains, strain designations, and culture conditions

Ninety-four *V*. *parahaemolyticus* clinical isolates, defined as isolated from a clinical (patient) source (e.g., wounds or stool), from 2010 to 2013 were provided by cooperating public health laboratories in MA, New Hampshire (NH), and Maine (ME) (Table 1). For the purposes of this study and in the absence of contradictory data, all these clinical isolates are considered pathogenic, regardless of genotype. Additional environmental strains from the Great Bay Estuary bordered by NH and ME prior to 2011 were also included for comparisons (Supplementary Table 1) (Mahoney et al., 2010; Ellis et al., 2012). A few previously unreported strains collected from the Great Bay Estuary in 2010–2013, and two environmental isolates from oysters harvested in CT that were recalled due to an outbreak were also used in this study (Supplementary Table 1). For the purpose of this study, we define environmental strains that lack hemolysin genes, and that are not phylogenetically related to strains from clinical sources as non-pathogenic. Environmental isolates that harbor either or both hemolysin genes, or are related to isolates from clinical sources (e.g., shared phylogeny or identical ST) are defined as potentially pathogenic. Finally, although not yet identified locally, isolates from the environment that are genetically indistinguishable from a clinical isolate by genotype and ST, and deemed clonal based on whole genome phylogeny are considered pathogenic. Several pathogenic *V. parahaemolyticus* isolates acquired previously included ST36 strain F11-3A (González-Escalona et al., 2008), pandemic strain MDOH-04-5M732 (Davis et al., 2007) and pre-pandemic strain BB22OP LM5132 (Mccarter, 1998), which were used as controls. Strains were grown in heart infusion (HI) medium with 3% NaCl (Fluka, Buchs, Switzerland) at 28°C (for environmental strains) or 37°C (for clinical strains) for routine culturing. Strains from environmental sources are enriched in APW, isolated on CHROMAgar Vibrio (CHROMagar, Paris, France) as purple colonies and cultured on T-soy agar as previously described (Schuster et al., 2011).

**Table 1:** **Sources and reporting states for clinical isolates available for this study**.

**Type of infection**	**Year**	**Strains by reporting state^1^**	**Potential exposure source traced to location^2^**
Gastric	2010	MAVP-E^a^	MA**^3^**
		MAVP-A, MAVP-T, NHVP-1	Unknown
	2011	MAVP-H^a^, MAVP-M^a^, MAVP-Q^a^	MA**^3^**
		MAVP-R^a^	MA
		MAVP-B, MAVP-C, MAVP-D^a^, MAVP-J, MAVP-K,MAVP-L, MAVP-N, MAVP-O^a^, MAVP-U^a^	Unknown
	2012	NHVP-2^a^	Unknown
	2013	MAVP-56^b^, MAVP-57^b^	Canada
		MAVP-7^b^	Canada, MA, or ME
		MAVP-55	Canada, MA, or other than North America
		MAVP-11^b^, MAVP-14^b^, MAVP-29^b^	Canada, MA, or WA
		MAVP-18^b^, MAVP-19^b^, MAVP-23^b^, MAVP-31^b^, MAVP-38^b^	CT
		MAVP-32^b^, MAVP-52^b^	CT or MA
		MAVP-44^b^	Canada, CT, or MA
		MAVP-40^b^	CT or VA
		MAVP-6^b^, MAVP-9^s^, MAVP-17^b^, MAVP-20^b^, MAVP-26^b^, MAVP-33^f^, MAVP-34^b^, MAVP-36^b^, MAVP-37^b^, MAVP-45^b^, MAVP-46^r^, MAVP-48^b^, MAVP-54^b^, MAVP-59^b^, MEVP-4^b^	MA
		MAVP-8^b^, MAVP-24^b^	MA, or ME
		MAVP-2^b^	MA, VA, or WA
		MEVP-2^e,d,i,l^, MEVP-6^c^	ME
		MAVP-3^p^	Other than North America
		MAVP-1^b^, NHVP-3^b^	VA
		MAVP-5^f^, MAVP-12^b^, MAVP-16^c,g^, MAVP-22^c^,MAVP-25^j^, MAVP-27^b^, MAVP-28, MAVP-30^b^, MAVP-35^f^, MAVP-39, MAVP-41^b^, MAVP-43^b^, MAVP-49, MAVP-50^b^, MAVP-51, MAVP-55^b^, NHVP-4^c^, MEVP-1^c,k^, MEVP-3^b,c,m,n,o^, MEVP-5^c,i,q^	Unknown
Wound	2011	MAVP-F, MAVP-G, MAVP-I, MAVP-X	MA
	2013	MAVP-13	MA
Unknown	2010	MAVP-P	Unknown
	2011	MAVP-S, MAVP-V, MAVP-Y	
	2012	MAVP-W	
	2013	MAVP-4, MAVP-10, MAVP-15, MAVP-21, MAVP-42, MAVP-47, MAVP-53, MAVP-58	

1 *Strains are coded by reporting state in the reference strain name and blinded by random assignment of letters (for MA isolates prior to 2013) or numbers (for all others); MAVP for MA, NHVP for NH, MEVP for ME. Potential exposure source(s) for each isolate is identified when reported as follows:^a^Oysters;^b^Raw oysters;^c^Lobsters;^d^Striped bass;^e^Cooked lobster;^f^Raw clams;^g^Fried clams;^h^Cooked clams;^i^Clams;^j^Quahogs;^k^Haddock;^l^Sea scallops;^m^Crab;^n^Shrimp;^o^Crawfish;^p^Sushi;^q^Fish chowder;^r^Handled bait;^s^Swallowed seawater while swimming*.

2 *Location where the V. parahaemolyticus contaminated food was harvested or where water exposure occurred. For wound infections, exposure presumed in reporting state*.

3 *Inferred that oysters potentially harvested from MA sources for these isolated cases reported from Cape Cod locations*.

### Genotypic analysis

Genotyping (Table 2, Supplementary Table 1) was performed by PCR amplification of template genomic DNA isolated from cultures grown in HI medium for 6 h using either Master Taq (5 PRIME, MD, US) or AccuStart PCR Supermix (Quanta, MD, US). DNA was extracted using the Wizard Genomic DNA Purification Kit (Promega, WI, USA), using columns and manufacturer-provided recipes (Epochlifescience Inc., TX, US), or by cetyltrimethylammonium bromide protein precipitation and organic extraction (Ausubel et al., 1990). The presence of a species-specific gene (*tlh*), both hemolysin genes (*tdh* and *trh*) and the pandemic marker ORF8 was determined using published primers and cycling parameters (Panicker et al., 2004) (Table 2, Supplementary Table 1). The presence of additional virulence-associated genes including a homolog of *Escherichia coli* cytotoxic necrotizing factor *vopC* as well as several additional genes located within pathogenicity islands of strain MDOH-04-5M732 including T3SS2 genes *vscC2* and effector *vopP*, was assessed using published primers and cycling parameters (Caburlotto et al., 2009). For each reaction, the presence of an amplicon of the correct size was determined following electrophoresis in 0.7% (for large amplicons) or 1.2% (for small amplicons) Sea Kem LE agarose (Lonza Group Ltd., NH, US) in 1× TAE buffer with 1× GelRed (Phenix Research Products, NC, US) for amplicon visualization. The size of each amplicon was determined by comparison to 1 Kb-plus ladder (Invitrogen Inc., NY, US) and also compared to amplicons from control strains including F11-3A and MDOH-O4-5M732. Presence of the correct size amplicon or gene (for sequenced strains) is denoted by (+), whereas absence of amplicon is denoted by (−).

**Table 2:** Distribution of genotypes^*^ among Northeastern US clinical isolates.

	***tdh*^a^**	***trh*^b^**	**ORF8^c^**	***vscC2*^d^**	***vop*P^e^**	***vop*C^f^**
**REFERENCE STRAINS BY SEROTYPE**						
O3:K6^G^	+	−	+	+	+	+
O4:K12^H^	+	+	−	−	−	−
**NUMBER OF NEW ENGLAND ISOLATES**						
2	+	−	+	+	+	+
2	+	−	−	+	+	+
2	+	−	−	−	−	−
3	−	+	−	+	−	−
1	−	+	−	−	−	+
4	−	+	−	−	−	−
1	+	+	−	+	−	−
66	+	+	−	−	−	−
2	−	−	−	+	−	−
11	−	−	−	−	−	−

* *Presence (+) or absence (−) of gene as determined by PCR*.

a Thermostable direct hemolysin.

b Thermostable-related hemolysin;

c O3:K6 Pandemic marker.

d Putative type III secretion system EscC protein. Chromosome II T3SS-pathogenic V. parahaemolyticus.

e Putative type III secretion effector YopP protein. Chromosome II T3SS-pathogenic V. parahaemolyticus.

f Homolog of E. coli cytotoxic necrotizing factor. Gene located on a pathogenicity island of V. parahaemolyticus.

G MDOH-04-5M732.

H *F11-3A*.

### Urease activity

Urease activity (Table 3, Supplementary Table 1) was determined on strains first grown in HI medium for 6 h at 28°C or 37°C, and then inoculated in triplicate as 10 μl samples onto 200 μl modified Christensen's urea agar, containing 2% NaCl, 0.1% peptone, 0.1% dextrose, 0.2% KH_2_PO_4_, 2% urea, 0.12% phenol red, and 2% agar in the wells of a 96-well plate. The plates were sealed with Breathe-Easy membrane (USA scientific Inc., FL, US) and incubated with ventilation at 37°C overnight. A positive reaction is observed as a change in color from yellow to pink. Wells without bacterial inoculum remained yellow. The association of the presence of a hemolysin gene (*tdh* or *trh*) and urease activity in isolates was determined using a two-tailed Fisher's exact test (Preacher and Briggs, 2001).

**Table 3:** **Correlation of urease activity with the presence of *trh***.

**Hemolysin genotyp**		**% of urease positive (# of strains tested)**
***tdh***	***trh***	
**FOR CLINICAL ISOLATES**		
+	+	100% (67)
+	−	25% (8)
−	+	100% (8)
−	−	33% (11)
**FOR ENVIRONMENTAL ISOLATES**		
+	+	100% (7)
+	−	0% (1)
−	+	100% (2)
−	−	0% (10)

### Genome sequencing and assembly

The *V. parahaemolyticus* ST631 strain MAVP-E, and four representative ST36 isolates including MAVP-26, MAVP-36, MAVP-45, all traced to shellfish harvest areas in MA, and MAVP-V, from an unknown source, were sequenced using an Illumina-HiSeq2500 device at the Hubbard Center for Genome Studies at the University of New Hampshire. Genomic DNA was extracted using the Wizard Genomic DNA Purification Kit (Promega, WI, USA) as recommended by some sequencing centers or by a cetyltrimethylammonium bromide and organic extraction method (Ausubel et al., 1990) that provides both higher quality and quantity of DNA but requires more technical skill. The DNA quality was assessed visually by electrophoresis. Sequencing libraries were generated from 1 μg of genomic DNA as determined using the Qubit 2.0 fluorimeter (LifeTech, CA, US). DNA was sheared on the Covaris M220 Ultasonicator to a mean size of 500 bp. Libraries were generated using the TruSeq Kit and targeted size selection of 500 bp was completed using the optional gel-extraction method in the TruSeq protocol (Illumina). MAVP-E was sequenced by a high output mode run, 101 bp paired-end whereas MAVP-26 and MAVP-36 were sequenced using a rapid output mode run, 150 bp paired-end with 152-fold coverage for MAVP-E, 249-fold coverage for MAVP-26, 238-fold coverage for MAVP-36, 355-fold coverage for MAVP-45, 847-fold coverage for MAVP-V, 84-fold coverage for MAVP-M, and 167-fold coverage for CT4287. Raw sequences were processed and *de novo* assemblies performed using the A5 pipeline (Tritt et al., 2012).

### Multi-locus sequence analysis and phylogenies, and analysis of population structure

**Figure 2:**
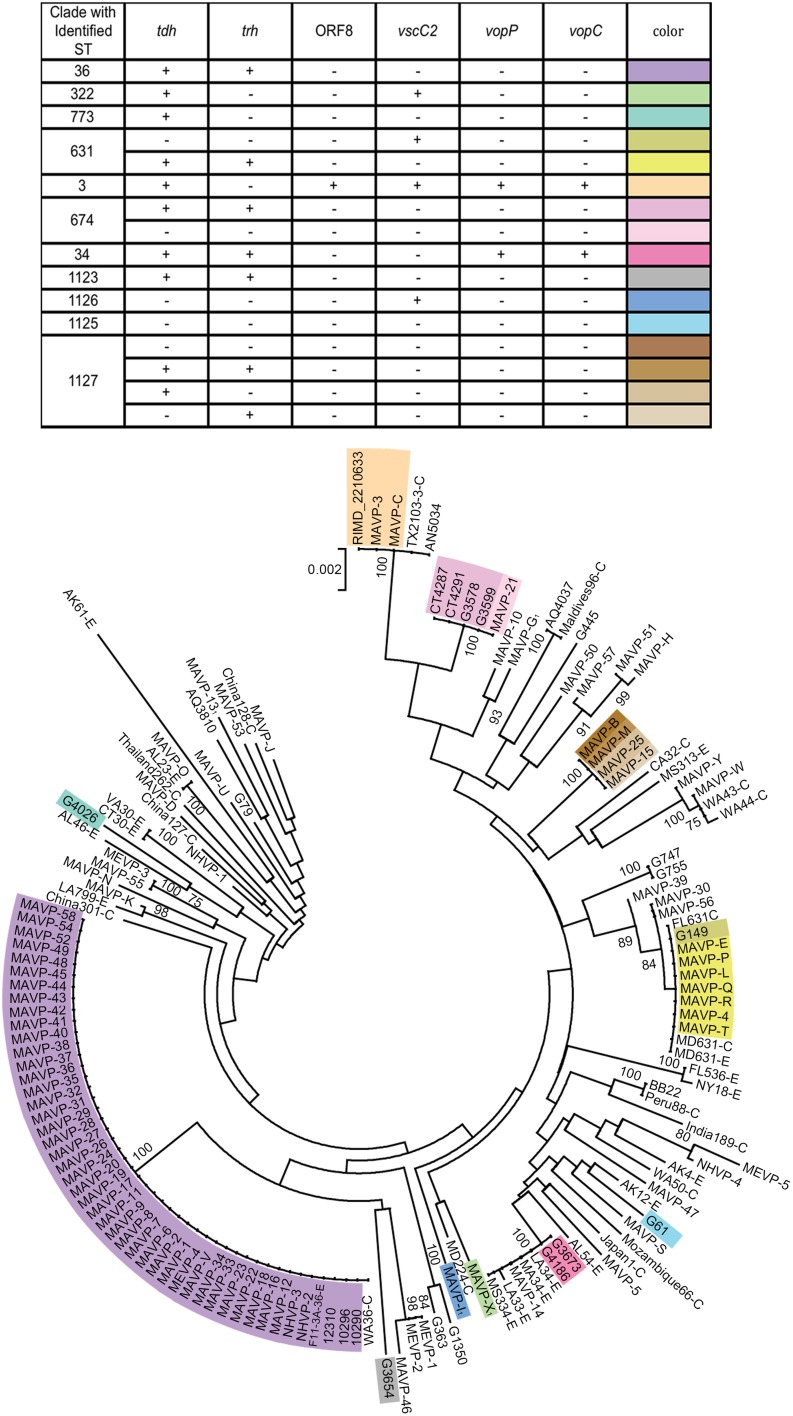
**Population structure of Northeast US clinical, related environmental, and some unique worldwide isolates of *V. parahaemolyticus***. A consensus neighbor-joining tree was constructed from four concatenated housekeeping gene loci including *dnaE, dtdS, pntA*, and *tnaA* sequences by using a Jukes-Cantor model. For ease in distinguishing pertinent information associated with worldwide strains from the MLST database, a single representative strain from several sequence types was identified from among the available strains, and the representative strain identified by geographic location (USA by state, international by country name), sequence type number, and as clinical (C) or environmental (E). Environmental strains from the region included isolates from the Great Bay Estuary (all with prefix G) or Connecticut (CT). For seven strains whose draft or complete genomes are publicly available, the loci were recovered from the available assemblies. Among related strains where the probable sequence type of the strains was determined, unique genotypes are indicated by color provided in the key and overlaid upon the tree. The bar indicates 0.2% divergences, and branches with less than 70% bootstrap support are unlabeled. Several clinical strains, for which one or more housekeeping loci were not successfully amplified and sequenced were excluded from the analysis (MAVP-A, MAVP-F, MAVP-59, MEVP-6). ^1^Isolates were from wound infections.

Phylogenetic analysis was performed from concatenated sequences derived by PCR amplification of multiple house-keeping loci. The amplicons were generated using Master Taq (5 PRIME, MD US), and sequenced by the Sanger method at the UNH Hubbard Center for Genome Studies or by Functional Biosciences (WI, US). For inferring multi-locus phylogeny, we used either seven loci (See Supplementary Figure 1) from two schemes as previously described (Ellis et al., 2012) including three loci adopted to determine the relationships broadly among *Vibrio* spp. (Sawabe et al., 2007) (*gyrB, pryH, and recA*) and four loci adopted to closely examine within species relationships (González-Escalona et al., 2008) (*dnaE, dtdS, pntA*, and *tnaA*); because these four are the only sequenced loci that overlap with those from strains in the public database (www.pubmlst.org), the phylogenetic relationships of a larger collection of isolates in this study with those of a global distribution were inferred using only four loci (*dnaE, dtdS, pntA*, and *tnaA*) (Figure 2). The primer sequences (Supplementary Table 2) and corresponding cycling parameters were used exactly as in published protocols (Sawabe et al., 2007; González-Escalona et al., 2008; Jolley, 2010). For phylogenies inferred from all seven loci, each forward and reverse raw sequence for 25 clinical isolates from 2010 to 2012 was assembled, and the contiguous sequences were then aligned and trimmed to match the length of corresponding sequence of 192 Great Bay Estuary environmental isolates (Ellis et al., 2012), only two of which harbor hemolysin genes. An additional eight isolates collected during 2013 were also included in some analysis. The sequences for individual isolates were then concatenated in alphabetic order. For phylogenies inferred from four loci (*dnaE, dtdS, pntA*, and *tnaA)*, each raw sequence was assembled, aligned, and trimmed to match the exact corresponding amplicon sequence from the public database. Neighbor-joining trees for concatenated sequence of either four loci (1868 bp) or seven loci (2988 bp) were constructed by Mega 5.0 software (Tamura et al., 2013) using Jukes-Cantor model. The statistical support was assessed by 1000 bootstrap re-assemblies.

Comparisons with the published MLST database (http://pubmlst.org) to identify STs were performed on 12 clinical and 16 environmental isolates for which the sequencing of three additional loci (*gyrB, pyrC*, and *recA*) were completed as described (González-Escalona et al., 2008). Raw sequences were assembled, aligned, and trimmed as described above. Allele numbers and ST numbers were determined by matching the public database. The STs of sequenced strains were determined from raw short read sequences using the short read sequence typing (SRST2) pipeline (Inouye et al., 2012).

The extent of recombination and mutation within the population was visualized and analyzed by several approaches. The contribution of recombination to phylogeny was evaluated visually using SplitsTree v4 neighbor net analysis of four loci, and the Phi test module was applied for determining statistical support (Huson and Bryant, 2006). The standardized index of association (I_A_^S^) was determined from a non-redundant allele database for the collection of 90 clinical and 16 environmental strains using the LIAN 3.5 linkage analysis program (Haubold and Hudson, 2000). This statistic describes the linkage disequilibrium in a multilocus data set where a low rate of recombination relative to mutation is indicative of linkage disequilibrium (I_A_ > 1). The null hypothesis that variation of the observed data (V_D_) does not differ from that predicted for a population in equilibrium (i.e., experiencing a high rate of recombination relative to mutation) (V_e_) was tested by a non-parametric Monte Carlo simulation, with the 5% critical value to determine significant linkage (*L*). ClonalFrame 1.1 was used to determine the relative influence of recombination compared to mutation (r/m) to nucleotide variation (Didelot and Falush, 2007).

### Reconstruction of whole genome phylogenies

Representative strains within the species *V. parahaemolyticus* were selected from among the 25 NCBI genome groups (defined as such by ~90% genome identity) from NCBI genomes phylogeny (http://www.ncbi.nlm.nih.gov/genome/691) that had accompanying information on geographic isolation, year, and sample source (environmental or clinical including wound, stool, and ear). The raw sequences from MAVP-E, MAVP-26, MAVP-36, MAVP-45, MAVP-V, MAVP-M, CT4287, (see Table 1 and Supplementary Table 1 for a description of these isolates) were processed and *de novo* assembled using the A5 pipeline (Tritt et al., 2012). The assembled contigs of all isolates were analyzed using REALPHY v. 1.09 (Bertels et al., 2014). Sequences were analyzed in three separate alignments, each with a unique reference strain including 10290 (GCA_000454205.1), BB22OP(NC_019955.1, NC_019971.1), and RIMD 2210633 (NC_004605.1, NC_004603.1), for phylogenies across a broad distribution of strains, and 10290, 10329 (NZ_AFBW01000001.1 - 33.1), and 10296 (GCA_000500105.1) for analysis of strains within the ST36 clonal complex clade. From these alignments multiple alignment positions were extracted and then merged into a single alignment. Neighbor-joining phylogenies were reconstructed using the maximum likelihood method in PhyML, with a GTR substitution matrix and a gamma-distributed rate heterogeneity model (Guindon et al., 2010). Phylogenies were visualized as trees using FigTree 1.4.2 (Rambaut, 2012). The branch length reflects nucleotide changes per by total number of nucleotides in the sequence.

### Environmental data and analysis

Sea surface water temperature (SST) data were acquired from US Integrated Ocean Observing System buoys in Long Island Sound and the Gulf of Maine (http://www.neracoos.org/datatools/climatologies) and from the NOAA National Estuarine Research Reserve System Wide Monitoring Program (SWMP) buoys in the Great Bay Estuary (http://cdmo.baruch.sc.edu/get/export.cfm). Monthly average SST data from 2004 to 2013 were compiled from representative buoys for the areas of interest. In Long Island Sound, the EXRX buoy is in close proximity to Norwalk Harbor, CT and Oyster Bay, NY, the AO1 buoy is representative of the southwestern end of the Gulf of Maine, and the Great Bay SWMP buoy is representative of the Great Bay estuary.

### Nucleotide sequences

MLST loci for strains, MAVP-I, MAVP-M, MAVP-E, MAVP-26, MAVP-36, G61, G3654, G4026, G3673, and CT4287 are available at www.pubmlst.org, and Illumina short read genomic sequences for bioproject PRJNA263814 including MAVP-26 (SAMN03107383), MAVP-36 (SAMN03107385), MAVP-E (SAMN03107386), MAVP-V (SAMN03177809), MAVP-M (SAMN03177808), CT4287 (SAMN03177811), and MAVP-45 (SAMN03177810) are available at NCBI.

## Results

### Genetic diversity among clinical isolates and distribution of *tdh, trh*, and urease activity

In light of the recent rise of *V. parahaemolyticus* infections in the northeastern US, clinical isolates from infections reported in MA, NH, and ME were obtained to identify pathogenic strain types, and determine whether they belong to resident or invasive lineages (See Table 1). A total of 94 clinical *V. parahaemolyticus* strains from infections reported from 2010 through 2013 were compared to each other and 200 environmental isolates from the region collected since 2007 (Mahoney et al., 2010; Ellis et al., 2012) (Table 1). Even though clinical isolates were not archived from every infection, and information was incomplete, this collection provides an extensive survey of regional infections (Table 1). Prior to 2013, consumption of local shellfish was implicated in only five, isolated infections (Table 1). However, by 2013 many clinical isolates (*n* = 37) were traced to at least one local source (Table 1 and see Newton et al., 2014). Gastric infections most often were attributed to contaminated oysters (*n* = 49), some of which were either definitively or potentially locally harvested (*n* = 27). In 2013, two infections were traced to recreationally harvested seafood in ME, including oysters, cooked fish, and lobster (Table 1). No illnesses were definitively traced to seafood from NH, although it is important to note that at the time of these collections commercial harvesting of oysters from this state was limited. Remarkably, two gastric infections were apparently induced by handling of raw product and casual ingestion of water while swimming, consistent with the presence of highly infective strains with a low infectious dose in near shore waters. Relatively fewer isolates (*n* = 5) were recovered from wound infections (Table 1).

The distribution of genotypes among clinical isolates indicated that infections were caused by a variety of strains, and although no specific gene or combination of virulence-associated genes defines pathogens reported in the region, certain genotypic profiles were more abundant than others (Table 2, and Supplementary Table 1). Very few isolates contained only the *tdh* gene (*n* = 6). Two isogenic, *tdh*-containing isolates, MAVP-C and MAVP-3, were identified as the pandemic O3:K6-type (Table 2, and Supplementary Table 1). Most clinical isolates from the Northeast US (80%) harbored the *trh* gene either alone or in combination with *tdh* (Table 2, Supplementary Table 1). In fact, strains harboring both *tdh* and *trh* were highly prevalent among clinical isolates from reported cases in this region even prior to 2013 (Table 1, and Supplementary Table 1). Yet several clinical isolates (*n* = 13), only three of which were wound isolates, harbored neither hemolysin.

Because urease activity may be useful for easily identifying some pathogenic types due to the proximity of the urease locus only ~7-Kb from the *trh* gene in the same pathogenicity island of some characterized strains (Park et al., 2000), we investigated whether urease activity correlated with the presence of hemolysin genes and clinical status among northern Northeast isolates (Tables 2, 3). Urease activity significantly correlated with the presence of *trh* (100% of isolates) either in the absence of *tdh* in clinical strains (*p* = 0.012) or in combination with the *tdh* gene for both clinical (*p* < 0.0001) and environmental (*p* < 0.0001) strains. Urease activity did not correlate significantly with *tdh* alone (*p* = 1.0). A few environmental isolates of unknown virulence were also urease positive, all of which harbor *trh* (Table 3).

### Phylogenetic relationships among clinical and environmental isolates and identification of resident clades

Neighbor-joining phylogenies identified shared lineages of environmental strains and pathogens isolated in the region (Supplementary Figure 1, Figure 2). Related environmental and clinical isolates were first identified by visual inspection of a neighbor-joining phylogeny based on seven genes sequenced from clinical strains isolated prior to 2013 (Supplementary Figure 1). This tree revealed that only eight out of 192 characterized environmental isolates were closely related to clinical isolates as inferred by bootstrap values greater than or equal to 70. The relationships of 90 clinical isolates, and 16 environmental (eight strains isolated prior to 2010, and eight previously unreported hemolysin-encoding strains isolated in 2013) to strains collected worldwide were then examined more broadly by MLSA using four loci shared with the public database (Figure 2). This analysis demonstrated that regional clinical isolates are polyphyletic and include some strains of known STs (Figure 2, Supplementary Table 3). Strains within clades are not always clonal when assessed using virulence-associated genotypes (Table 2, Supplementary Table 1, Figure 2).

Even though definitive traceback data was incomplete prior to 2013, the reporting data (see Table 1) did facilitate identification of the isolates most likely from local sources, and those from outside the region. Both isolates harboring ORF8 grouped with the globally distributed pandemic ST3 strains, but these were not from local sources. The ST631 clade grouped isolates from multiple years that were traced to oysters consumed at various Cape Cod MA locations where oysters are farmed and imported oysters are not typically marketed and thus they were identified as putatively local, and a single isolate was traced to a local source (Table 1, Figure 2). A single environmental ST631 isolate (G149) that based on whole genome phylogeny is a close relative to these strains (Xu, unpublished data) was also isolated in NH in 2007. In agreement with the persistence of this strain type, ST631 are not strict clones (Supplementary Table 1, Figure 2). MAVP-14, and two environmental isolates from NH harboring both *tdh* and *trh* grouped with several ST34 environmental strains from the Gulf of Mexico, specifically Louisiana (LA) (González-Escalona et al., 2008) (Supplementary Table 3). MAVP-21 was most closely related to four *tdh*^+^/*trh*^+^ ST674 environmental isolates, two of which were recovered from oysters harvested from CT and two from the Piscataqua River of the GBE (Figure 1). Finally, even though genetically diverse strains identified as a new ST, ST1127, caused four infections, three were from unknown sources and no environmental isolates of this ST have been identified, thus it is not clear whether they are residents.

**Figure 3:**
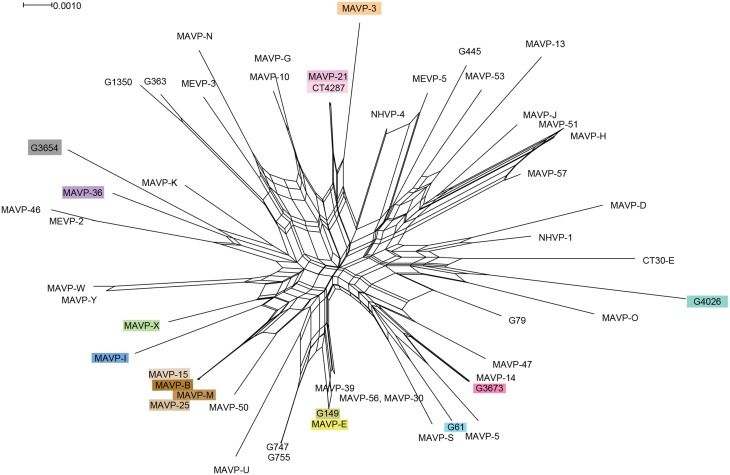
**Recombination network revealed by splits decomposition analysis of genotypes of Northeast regional *V. parahaemolyticus* isolates**. The Splits Tree analysis is based on four concatenated gene loci including *dnaE, dtdS, pntA*, and *tnaA* (1868 bp) from 54 unique regional clinical and related environmental isolates. Where multiple strains were identified as sharing the same allelic profile or ST, one representative strain per unique genotype was selected for inclusion in analysis. For ease in identifying resident and invasive clades, strains are designated by colors that correspond exactly to color scheme in Figure 2.

We evaluated the MLSA data by several statistical tests to determine the relative contributions of mutation and recombination to population structure. The influence of recombination on clonal structure was supported by the Phi test in SplitsTree v.4 (*P* < 0.001), and was also indicated by the reticulate structure of the Neighbor Net analysis (Figure 3). However, the LIAN test of recombination indicated the population is in linkage disequilibrium (*P* < 0.01; *L* = 0.1009), with a standardized index of association of I_A_^S^ = 0.2228, which is also visible in the Neighbor Net analysis as long branch lengths emerging from a central network of recombination. Approximately one recombination event for every three mutations is predicted (r/m = 0.337707; 95% credibility region = 0.145408 – 0.571994), which indicates an effect of both recombination and mutation upon population structure.

### Occurrence of non-indigenous ST36 clinical isolates in the northeast US

Most clinical isolates from 2013 (*n* = 42) were closely related to each other and to ST36 clonal complex strains from the PNW (Figure 2) (Newton et al., 2014). The ecological invasion of the ST36-clade strain in the Atlantic from the Pacific was suggested when these genotypes were associated with infections reported in NY in 2012 (Martinez-Urtaza et al., 2013). All isolates from the northern New England region in this ST36-clade shared the same virulence associated genotype; however, MAVP-V of unknown source and that was isolated in 2011, is distinct from the others from the Northeast in that it is missing a phage-encoding island that is also missing in closely related strain 12310 from Washington state (Haendiges et al., 2014) (Table 1, Supplementary Table 1, Figure 2, and Supplementary Figure 1). Remarkably, the ST36-clade isolates were not only traced to oysters harvested south of Cape Cod proximal to Long Island Sound and Oyster Bay NY, but also from north of Cape Cod in the Gulf of Maine (See Figure 1). This geographical distance suggests that the New England ST36-clade strains both spread northward and grew sufficiently to increase infections (Newton et al., 2014). MAVP-26, MAVP-36, and MAVP-45 (traced to at least two, and potentially three shellfish harvest areas in MA), and MAVP-V (from an unknown source) were subsequently confirmed to be ST36 by whole genome sequencing.

### Whole genome phylogeny of *V. parahaemolyticus* and relationships between ST36 atlantic and pacific populations

**Figure 4:**
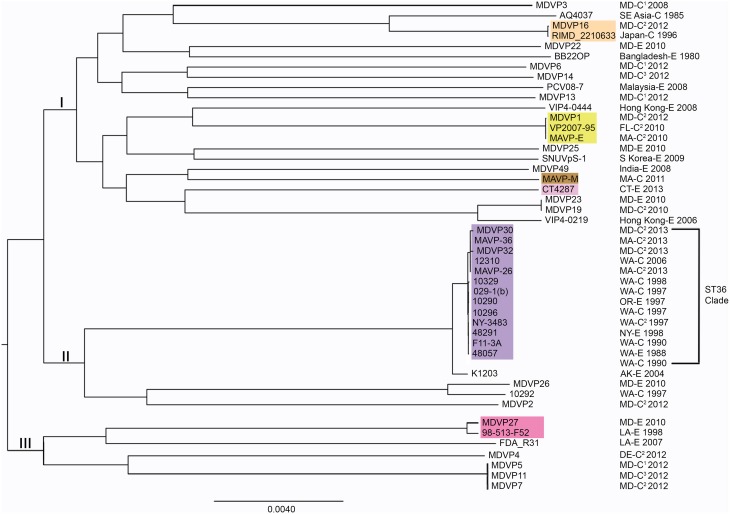
**Phylogenies of pathogenic lineages of *V. parahaemolyticus***. Multiple genome reference-sequence alignment based phylogeny were reconstructed using REALPHY v1.09 to evaluate the evolution and relatedness of pathogenic lineages, where related pathogens grouped within three major nodes (I, II, and III). Maximum likelihood phylogenies of strains of broad phylogenetic distribution were reconstructed based on merged reference strains 10329, BB22OP, and RIMD 2210633, where the merged alignment represents 63% coverage of sites of the largest reference genome (Vp10329). The branch length reflects nucleotide changes per by total number of nucleotides in the sequence. Representative strains are identified by geographic location (USA by state, international by country name), as clinical (C) or environmental (E) and year isolated. For ease in identifying strains or sequenced types identified in the Northeast, select strains are designated by colors that correspond exactly to color scheme in Figure 2. ^1^Isolates were from wound infections. ^2^Isolates were from gastric infections. ^3^Isolates were from ear infections.

From the collection of available draft genomes of strains of known identity (i.e., clinical and environmental source) and geographic location of isolation, we examined phylogenetic relationships among isolates of *V. parahaemolyticus*. Although the selected strains likely underrepresent the diversity and distribution of pathogen types, they nevertheless provide insight into the evolution of different lineages. Distinctive pathogen lineages grouped within three major nodes, one of which included the pandemic RIMD 2210633, BB22OP, and northern New England resident ST631 clades, a second of which grouped the ST36 clade and other clinical and environmental strains from both the Atlantic and Pacific, and a third that grouped fewer and more distantly-related representative strains from the Atlantic and Gulf of Mexico (Figure 4). This broad phylogenetic relationship illustrates that although infectious strains of *V. parahaemolyticus* are polyphyletic, they may yet belong to genetic clusters that can be diagnostically and epidemiologically informative.

**Figure 5:**
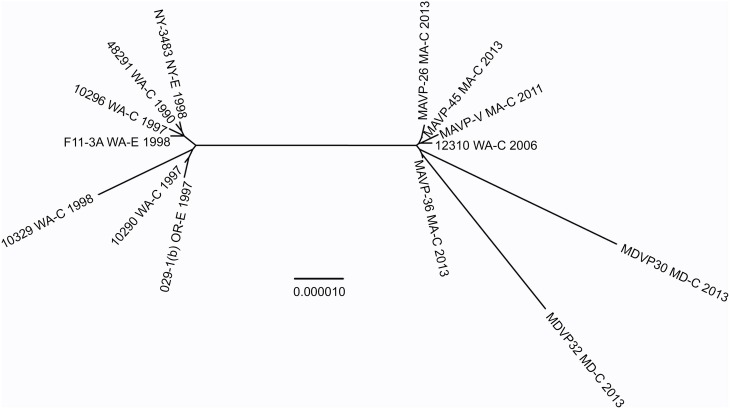
**Phylogenetic relationship between Atlantic and Pacific ST36 populations**. Multiple genome reference-sequence alignment based phylogeny were reconstructed using REALPHY v1.09 to evaluate the evolution and relatedness of ST36 pathogenic lineages. Maximum likelihood phylogenies within the ST36 clade were reconstructed based on merged reference strains 10329, 10290, and 10296 where the merged alignment represents 92% coverage of sites of the largest reference genome (Vp10329). The branch length reflects nucleotide changes per by total number of nucleotides in the sequence. Representative strains are identified by geographic location [USA by state, as clinical (C) or environmental (E) and year isolated].

To test this hypothesis, the genomes and relationships of related ST36-clade strains were compared to gain insight into the patterns of microevolution within this clade (Figure 5). As expected from a dynamic of recent ecological invasion in the Atlantic, most strains within this clade isolated prior to 2011 were from the PNW, where this strain is indigenous. One exception was a single environmental isolate from NY in 1998 NY-3483. NY-3483 grouped with these historic PNW ST36 strains. More recent clinical isolates from 2013 traced to Atlantic sources were closely related to and share a common ancestor with strain 12310, a Washington State (WA) isolate from 2006. This topology strongly supports that the Atlantic ST36 strains are non-indigenous, and were derived from the PNW ST36 population.

## Discussion

*V. parahaemolyticus* has become the most common bacterial infection acquired from seafood in the US, with an estimated 35,000 cases each year (Scallan et al., 2011). Even with protective measures in place in the Gulf States, and the PNW, reported cases continue to increase nationwide (Crim et al., 2014). But disease burden in the northeastern US had until recently remained relatively low, even with these national trends. Over the last several years, assumptions that pathogens could not thrive in the cooler waters of the Northeast have given way as several unprecedented outbreaks occurred across multiple states leading to the implementation of protective measures in both CT and MA. The water temperatures in the Long Island Sound and the Gulf of Maine were at or near 11-year maxima from October 2011 through September 2012, and again in July 2013 (Dawicki, 2013; NERACOOS, 2014) (Figure 1). The first outbreaks coincided with these unusually mild conditions that have also had profound effects on the Gulf of Maine ecosystem (Mills et al., 2013). Likewise, many previous US outbreaks and the spread of the pandemic strain type on the Pacific US coast have also been linked to warmer ocean temperatures (Mclaughlin et al., 2005; Martinez-Urtaza et al., 2008), a predicted effect of global climate change, indicating that these recent outbreaks in the Northeast US may herald a continuing trend of increased disease in this region, and for others in more northern latitudes (Baker-Austin et al., 2013).

The increase in recent infections in the Northeast was associated with both resident and non-indigenous *V. parahaemolyticus* lineages. The establishment of the ST36 strain, first reported in the region during an outbreak in Oyster Bay NY in 2012 explains the abrupt increase in cases traced to several different locations of northern New England in 2013, including one location in CT proximal to Oyster Bay NY, and two geographically distant locations in MA, to the south and north of Cape Cod (Martinez-Urtaza et al., 2013; Newton et al., 2014) (See Results Section – Occurrence of Non-Indigenous ST36 Clinical Isolates in the Northeast US, Figure 1, and Table 1). However, even before these unprecedented multi-state outbreaks, cases were on the rise in the region from potentially emergent residential lineages including ST631, ST34, and ST674 (See Table 1, Supplementary Figure 1, and Figure 2). Genetically diverse ST631 strains were responsible for seven isolated infections between 2010 and 2013 (Figures 1, 2, Table 1, Supplementary Figure 1). Previously identified pathogenic ST631 isolates have been traced to Florida (FL) (Noriea Iii et al., 2010), and Maryland (MD) (Haendiges et al., 2014) indicating a fairly wide Atlantic US-coastal distribution of this ST (Supplementary Table 3). The identification of a single environmental ST631 isolate from NH (G149) lacking *tdh* and *trh*, that was closely related to the clinical isolates based on whole genome maximum likelihood phylogeny (Xu and Whistler, unpublished) indicates the clade is resident rather than transient in New England (Ellis et al., 2012). A single clinical isolate potentially from a local oyster and two environmental isolates were identified as ST34 (See Table 1, Figure 2). Although environmental ST34 isolates of unknown virulence are broadly distributed from the Pacific and Gulf of Mexico waters, two previously identified ST34 isolates are from clinical sources, indicating the potential for virulence among this clade (Supplementary Table 3) (González-Escalona et al., 2008). Finally, within the ST674 lineage, a single clinical isolate from an unknown source and four environmental isolates from CT and NH oysters were identified (Table 1, Figure 2). These are the first ever reported strains of this ST and their presence in more than one location in New England suggests these related strains are resident and potentially endemic. Further analysis of the clinical and environmental strains from these resident lineages may provide insight into evolution and recent emergence of pathogenic types in the region.

The recent appearance of ST36-clade strains in the Atlantic is apparently not the first invasion: one isolate, NY-3483, was identified in NY in 1998, when one of the largest multi-state outbreaks of *V. parahaemolyticus* in the US occurred in NY and CT (CDC, 1999). That outbreak was attributed to the invasive pandemic O3:K6 strain type, not the ST36 strain type. This coincidence could indicate that certain conditions promoted the ecological invasion of non-resident pathogenic strain types, but neither strain was apparently able to establish residency. The whole genome phylogeny of these ST36 strains indicates that current ST36 strains causing infections in the Northeast are not derived from NY-3483 (Figure 5). Rather, the current ecologically invasive population shares a common ancestor with a quite distinctive 2006 isolate from the PNW (Figure 5). Curiously, the serologically unique ST36 strains responsible for several outbreaks in the PNW in 2006 (both WA and Canada) did not persist in that region (Banerjee et al., 2014). Furthermore, current trends in the PNW suggest that infections by ST36 strains have declined locally for as yet unknown reasons (http://www.cdc.gov/vibrio/investigations/). In the Atlantic, the non-indigenous ST36 lineage has persisted for several years, and spread northward into the Gulf of Maine (Figure 1). This strain recurrence suggests that yet-undefined environmental factors, perhaps in combination with a particular genetic predisposition, allowed it to compete with resident *V. parahaemolyticus* strains. Furthermore, two unique MD ST36 isolates chosen for this analysis for comparison (Haendiges et al., 2014) also share a common ancestor with the MA isolates (and more specifically MAVP-36), suggesting the clinical populations in the Northern and Mid-Atlantic could undergo admixture. However, the greater genetic distance of the MD isolates from other strains could reflect subsequent evolution following an earlier introduction or could indicate more rapid reproduction for this subpopulation as would be anticipated in the warmer ocean waters compared to the Northeast (Figure 5).

The analysis of the contributions of mutation and recombination indicates a significant effect of both processes upon population structure (Figure 3) (Vos and Didelot, 2009). The reticulate nature of the SplitsTree phylogeny is consistent with multiple evolving subpopulations of pathogens undergoing recombination, but not frequently enough to break up the distinct population structure of the major lineages (Figure 3) (Huson and Bryant, 2006). This data and its interpretation may appear to contrast with those inferred from other *Vibrio* sp. for which mutation is lower, not higher than recombination (Schuster et al., 2011; Turner et al., 2013); however, the subpopulations under scrutiny are often more closely related, and sometimes exclude environmental counterparts. When isolates that were not traced specifically to Northeast sources were excluded from this analysis, the population structure of regional isolates agreed with other studies that indicate substantial recombination among *V. parahaemolyticus* (González-Escalona et al., 2008; Ellis et al., 2012) (Figure 3). As one example, the SplitsTree analysis indicates a striking and more recent recombination history among ST631 and related non-ST631 environmental and clinical strains that could have influenced the evolution and potentially the emergence of this and new pathogenic types in the region (Figure 3). In contrast, the Northeast ST36 population has evidently evolved primarily through mutation, but this recent population expansion likely has provided insufficient time for the effects of recombination to be apparent. Even so, several resident strain lineages from local sources, including G3654, MAVP-46, and MEVP-2, were identified as potentially having a past recombinatorial history with the ST36 lineage.

Pathogenic strains from the Northeast, even those that are resident, are genotypically diverse (Table 2, Supplementary Table 1, Figure 2), making the development of an optimal detection strategy for all pathogens extremely challenging. The majority of infections in the Northeast were caused by *trh*-containing, urease positive strains (Tables 2, 3, Supplementary Table 1), and both attributes are used for assignment of environmental isolates as pathogens since *trh*-containing strains have increased among clinical isolates in North America in recent years (Tables 2, 3) (Jones et al., 2012; Martinez-Urtaza et al., 2013; Banerjee et al., 2014). Utility of urease activity as a surrogate for *trh* detection in Northeast *V. parahaemolyticus* populations is well supported by our analysis (Table 3). Yet the reliability of *trh* as a virulence marker for environmental surveillance is not thoroughly validated because the pathogenic potential of environmental isolates with this trait from any coastal population is still untested. Directed experimentation must determine whether environmental isolates that harbor hemolysins are in fact virulent and, conversely, that those lacking these genes are not virulent. However, virulence studies with environmental isolates are uncommon and reveal potential shortcomings in virulence models currently in use (Caburlotto et al., 2009; Mahoney et al., 2010). For instance, Mahoney et al. examined the cytotoxicity, a commonly-used proxy for virulence, of a variety of known pathogenic strains compared to environmental isolates lacking any of the classic virulence determinants used in surveillance, including *tdh, trh*, and T3SS2 (Mahoney et al., 2010). Surprisingly, many environmental isolates that would otherwise be identified as non-pathogenic are more cytotoxic to human CaCo-2 cells than most clinical strains, indicating relative cytotoxicity as a measurement for virulence may only be useful for studies comparing known pathogens, and not for environmental isolates (Mahoney et al., 2010). Thus, the determination of virulence potential and assignment of an environmental isolate as a pathogen is not an easy task given the limitations of current models for disease and infection. Ultimately, a better understanding of the prevalence of any potential diagnostic trait among non-pathogens in the environment is necessary.

The current state of knowledge supports multiple points of view on the utility of hemolysins as a diagnostic trait, even with their strong correlation with clinical isolation (Nishibuchi et al., 1992; Honda and Iida, 1993; Xu et al., 1994; García et al., 2009; Hiyoshi et al., 2010; Thongjun et al., 2013; Banerjee et al., 2014). It is concerning that 14% of clinical isolates, 11% if counting those from gastric infections only, harbored neither hemolysin gene (Table 2), and therefore, these strains would evade detection as human pathogens in any monitoring program relying upon only these markers. A similar prevalence of clinical strains lacking these virulence markers has been observed in other regions of North America (Jones et al., 2012; Banerjee et al., 2014). One explanation for the lack of *tdh* or *trh* in these stool isolates evokes the possibility of misidentification of non-pathogens consumed along with pathogens in an oyster or other food, since standard practices limit analysis to only one isolated colony in confirmed laboratory tests. However, we see this as improbable given that such isolates would need to both colonize and proliferate in order to achieve a high enough population to allow detection at this relatively high frequency. Furthermore, the detection of the same strain repeatedly during an outbreak would be unlikely if other, non-pathogenic *V. parahaemolyticus* were so abundant during infection. Even though published reports support that hemolysins contribute to virulence and are sufficient for some symptoms (Nishibuchi et al., 1992), other studies demonstrate virulence is unaffected by the deletion of these genes (Xu et al., 1994; Park et al., 2000). Thus this relationship is unresolved and supports the view that non-hemolysin producers isolated from humans may be pathogenic. More thorough analyses of the diversity of isolates from single infections are necessary to address this limitation in our knowledge of the defining characteristics of pathogens. If non-hemolysin producers are pathogenic, analysis of their genomic attributes may provide useful insight into fundamental attributes that promote their virulence. Even in the absence of a definitive (or even a few) virulence marker(s) harbored by all pathogenic *V. parahaemolyticus* strains, there is promise for monitoring particular lineages of concern (see Figure 2), by the use of markers or pathogenicity islands identified by whole genome comparisons. Assays informed by genomics comparisons could be tailored to different regions, but would need to include new strain diagnostic loci as populations continue to evolve and are influenced by invasive and introduced strains. Ultimately, the above limitations suggest that a combined trait or multi-locus-based assessment that includes hemolysins may be necessary for monitoring how changing population structure correlates with increased disease incidence, and for assessing public health risk.

## Conclusions

Environmental conditions that create a warmer and longer season conducive for rapid growth do not entirely explain the current trend of increasing numbers of *V. parahaemolyticus* infections from local northern New England sources. This study suggests that changes in the bacterial populations underlie enhanced disease risk in the region. Yet, other factors may also contribute to increases in the reported number of cases. First, commercial shellfish harvesting has risen steeply in both MA and CT in recent years, with overall increased summer harvesting and consumption of product from the area (MADMF, 2013). But this increase does not explain the rise in cases in ME from recreationally harvested shellfish. Second, Vibriosis is now a reportable disease in all New England states, and an increased awareness of the pathogen by the public and health practitioners may contribute to the rise in reported cases. Regardless, the status of *V. parahaemolyticus* as an emergent pathogen of significant concern in the region is justified by projections of changing climate that may be conducive to *V. parahaemolyticus* growth. This concern is amplified by the finding that a non-indigenous pathogen invaded the Atlantic during a period with warmer than usual ocean temperature and has now likely established populations in the Northeast (Newton et al., 2014). Since *Vibrio* species are known to undergo recombination readily (Turner et al., 2013), and recombination influenced the population structure of these isolates (Figure 3) it is not yet clear how the now likely-established population of the Atlantic ST36 strain could further shape the Northeast resident pathogen population. Our findings lay a foundation for future research aimed at understanding the interplay between pathogen genotype and environment that leads to disease emergence.

### Conflict of interest statement

The authors declare that the research was conducted in the absence of any commercial or financial relationships that could be construed as a potential conflict of interest.
