# Supplementary material for: Genetic characterization of clinical and environmental Vibrio parahaemolyticus from the Northeast USA reveals emerging resident and non-indigenous pathogen lineages
Source: Front Microbiol. 2015 Apr 7;6:272. doi: 10.3389/fmicb.2015.00272 (PMC4387542; doi:10.3389/fmicb.2015.00272)
Supplement: Supplementary file 1 [file DataSheet1.PDF]

Supplementary Material

Genetic characterization of clinical and environmental *Vibrio parahaemolyticus* from the Northeastern US reveals emerging resident and non-indigenous pathogen lineages

Feng Xu[1, 2, 3], Saba Ilyas[1], Jeffrey A. Hall[1, 3], Stephen H. Jones[3, 4], Vaughn S. Cooper[1, 3] and Cheryl A. Whistler[1, 3]*

[1] Department of Molecular, Cellular and Biomedical Sciences, University of New Hampshire, Durham, New Hampshire
[2] Graduate Program in Genetics, University of New Hampshire, Durham, New Hampshire
[3] Northeast Center for Vibrio Disease and Ecology, University of New Hampshire, Durham, New Hampshire
[4] Department of Natural Resources and the Environment, University of New Hampshire, Durham, New Hampshire
*** Correspondence:** Corresponding author, Department of Molecular, Cellular and Biomedical sciences, University of New Hampshire, 46 College Rd., Durham, NN, 03824, USA
E-mail: cheryl.whistler@unh.edu

Supplementary Data

1. Supplementary Figures and Tables

1.1. Supplementary Tables

Supplementary Table 1. Strains with relevant pathogenicity markers and urease activity[a]

Strains	*tdh*[b]	*trh*[c]	ORF8[d]	*vscC2*[e]	*vopC*[f]	*vopP*[g]
Reference strains						
MDOH-04-5M732[a]	+	-	+	+	+	+
BB22[a]	+	-	-	+	+	+
F11-3A	+	+	-	+	+	+
Clinical isolates from northern New England						
MAVP-3[a], MAVP-C[a]	+	-	+	+	+	+
MAVP-D[a], MAVP-F	+	-	-	+	+	+
MAVP-25, MAVP-57[a]	+	-	-	-	-	-
MAVP-G, MAVP-J, MAVP-N	-	+	-	+	-	-
MAVP-K	-	+	-	-	-	+
MAVP-S, MAVP-14, MAVP-15, MAVP-51	-	+	-	-	-	-
MAVP-O	+	+	-	+	-	-
MAVP-A, MAVP-E MAVP-H, MAVP-L, MAVP-M, MAVP-R MAVP-P, MAVP-Q, MAVP-T, MAVP-U, MAVP-V, MAVP-W, MAVP-Y, MAVP-1, MAVP-2,	+	+	-	-	-	-

MAVP-4, MAVP-6, MAVP-7, MAVP-8, MAVP-9, MAVP-11, MAVP-12, MAVP-16, MAVP-17, MAVP-18, MAVP-19, MAVP-20, MAVP-22, MAVP-23, MAVP-24, MAVP-26, MAVP-27, MAVP-28, MAVP-29, MAVP-30, MAVP-31, MAVP-32, MAVP-33, MAVP-34, MAVP-35, MAVP-36, MAVP-37, MAVP-38, MAVP-39, MAVP-40, MAVP-41, MAVP-42, MAVP-43, MAVP-44, MAVP-45, MAVP-46, MAVP-48, MAVP-49, MAVP-50, MAVP-52, MAVP-54, MAVP-56, MAVP-58, NHVP-2, NHVP-3, MEVP-1, MEVP-2, MEVP-3, MEVP-4, MEVP-5, MEVP-6						
MAVP-I[a], MAVP-X[a]	-	-	-	+	-	-
MAVP-B, MAVP-5[a], MAVP-10[a], MAVP-13[a] MAVP-21[a], MAVP-47, MAVP-53[a], MAVP-55, MAVP-59[a], NHVP-1[a], NHVP-4[a]	-	-	-	-	-	-
Environmental isolates from New England						
G4026[a]	+	-	-	-	-	-
G363, G1350	-	+	-	-	-	-
G3673, G4186	+	+	-	-	+	+
CT4287, CT4291, G3654, G3578, G3599	+	+	-	-	-	-
G149[a]	-	-	-	+	-	-
G61[a], G79[a] G747[a], G755[a]	-	-	-	-	-	-

[a] Denotes strains that don't exhibit urease activity.
[b]Thermostable direct hemolysin
[c]Thermostable-related hemolysin
[d]O3:K6 Pandemic marker
[e]Putative type III secretion system EscC protein. Chromosome II T3SS-pathogenic *V. parahaemolyticus*
[f]Putative type III secretion effector YopP protein. Chromosome II T3SS-pathogenic *V. parahaemolyticus*
[g]Homolog of *E. coli* cytotoxic necrotizing factor. Gene located on a pathogenicity island of *V. parahaemolyticus*

Supplementary Table 2. Oligonucleotide primers used in this study

Gene /locus	Primer sequence (5'-3')	Amplicon size (bp)	Source
tlh	F: AAAGCGGATTATGCAGAAGCACTG<br>R: GCTACTTTCTAGCATTTTCTCTGC	450	Panicker et al.,2004
tdh	F: GTAAAGGTCTCTGACTTTTGGAC<br>R: TGGAATAGAACCTTCATCTTCACC	269	Panicker et al.,2004
trh	F: CATAACAAACATATGCCCATTTCCG<br>R: TTGGCTTCGATATTTTCAGTATCT	500	Panicker et al.,2004
ORF8	F: AGGACGCAGTTACGCTTGATG<br>R: CTAACGCATTGTCCCTTTGTAG	369	Panicker et al.,2004
vscC2	F: GCGGTCTATTGCTATCCT<br>R: TCTTGGTATTGATAGTGGGTG	362	Caburlotto et al.,2009
vopP	F:CGTCCAACTCTATTGTTGTG<br>R: CAATGTTGGCTATTCGGTTG	393	Caburlotto et al.,2009
vopC	F: CAGAGTTGGTTTCGCAG<br>R: CTGGTACGCCTCTTGGACAG	579	Caburlotto et al.,2009
dnaE	F: CGRATMACCGCTTTCGCCG<br>R:GAKATGTGTGAGCTGTTTGC	596	Chowdhury et al.,2004
dtdS	F: TGGCCATAACGACATTCTGA<br>R: GAGCACCAACGTGTTTAGC	497	Chowdhury et al.,2004
gyrB	F: GAAGGBGGTATTCAAGC<br>R: GAGTCACCCTCACWATGTA	629	Chowdhury et al.,2004
gyrB-1	F: GAAGTTATCATGACGGTACTTC<br>R: CCTTTACGACGAGTCATTTC	900	Sawabe et al.,2007
pntA	F: ACGGGCTACGCAAAAGAAATG<br>R: TTGAGGCTGAGCCGATACTT	470	Chowdhury et al.,2004
pyrC	F: AGCAACCGGTAAAATTGTCG<br>R:CAGTGTAAGAACCGGCACAA	533	Chowdhury et al.,2004
pyrH	F: GATCGTATGGCTCAAGAAG<br>R: TAGGCATTTTGTGGTCACG	450	Sawabe et al.,2007
recA	F: GAAACCATTTCAACGGGTTC<br>R: CCATTGTAGCTGTACCAAGCACCC	773	Chowdhury et al.,2004
recA-1	F: GTCTACCAATGGGTCGTATC<br>R: GCCATTGTAGCTGTACCAAG	600	Sawabe et al.,2007
tnaA	F: TGTACGAAATTGCCACCAAA<br>R: AATATTTTCGCCGCATCAAC	463	Chowdhury et al.,2004

Supplementary Table 3. Sequence types (ST) of northern Northeast US environmental isolates previously isolated in other locations

Isolate	Name	ST	Source*	Region /Country	Serotype	TDH/TRH
43	98-513-F51	34	E	LA (1998)	O4:K9	+/+
44	98-513-F52	34	E	LA (1998)	O4:K9	+/+
45	98-548-D11	34	E	MA(1998)	O4:K9	+/+
187	224	34	C	Norway	O3: Kunk	+/+
272	106E1	34	E	LA(2006)	Unknown	+/+
290	2041 Fc2r2	34	E	LA (2007)	Unknown	+/+
295	2211Ta2r1 1	34	E	LA(2007)	Unknown	+/+
297	2441Ea r1 1	34	E	LA(2007)	Unknown	+/+
300	2441Za r210	34	E	LA(2007)	Unknown	+/+
301	2441Zb r1 4	34	E	LA(2007)	Unknown	+/+
706	Vp-2007-042	34	E	MS 8-11C(2007)	Unknown	+/+
846	27	34	E	WA(2007)	Unknown	+/+
852	50	34	E	WA(2007)	Unknown	+/+
856	260	34	E	WA(2007)	Unknown	+/+
866	765	34	E	WA(2007)	Unknown	+/+
1280	Vp27julie	34	E	USA(2010)	Unknown	Unknown
1380	50	34	C	USA(2006)	Unknown	Unknown
1271	Vp1	631	C	MD(2012)	Unknown	+/+
1286	Vp31	631	C	MD(2013)	Unknown	+/+
1290	Vp35	631	E	MD(2013)	Unknown	+/+
1346	VP2007-095	631	C	FL(2007)	Unknown	+/+

Isolate ID is from www.pubmlst.org; *Environmental (E) and clinical (C) designated.

1.2. Supplementary Figures

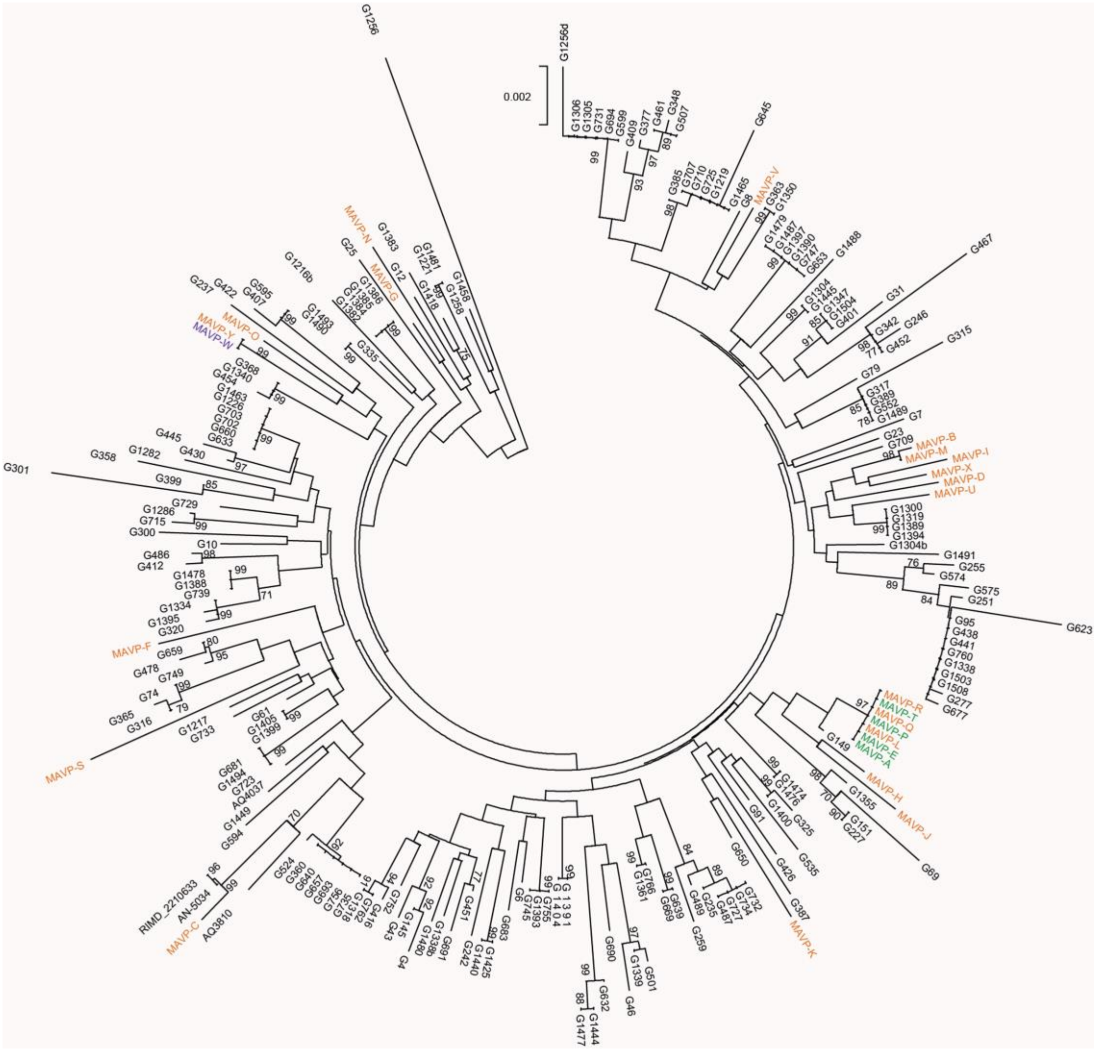

Supplementary Figure 1. Population structure of clinical isolates from MA from 2010-2012 and related NH environmental isolates. A consensus neighbor-joining tree was constructed from seven concatenated housekeeping gene loci including *gyrB*, *pyrH*, *recA*, *dnaE*, *dtdS*, *pntA*, and *tnaA* by using a Jukes-Cantor model. The statistical support was assessed by 1,000 bootstrap re-assemblages. Green: MA clinical strains isolated from 2010 Orange: MA clinical strains isolated from 2011 Purple: MA clinical strains isolated from 2012.
